# Development of a curriculum for interdisciplinary e-learning on delirium in nursing homes—a modified Delphi study

**DOI:** 10.1186/s12909-025-07078-x

**Published:** 2025-04-07

**Authors:** Vincent Molitor, Johanna Christina Seiters, Horst Christian Vollmar, Rebecca Palm

**Affiliations:** 1 School VI - School of Medicine and Health Sciences, Carl Von Ossietzky University, Oldenburg, Germany; 2 Institute of General Practice and Family Medicine (AM RUB), Medical Faculty, Ruhr University Bochum, Bochum, Germany

**Keywords:** Delphi study, Curriculum development, E-Learning, Delirium, Nursing home, Health care professionals

## Abstract

**Background:**

Health care professionals (HCPs) in nursing homes, such as nurses and general practitioners (GPs), indicate a need for delirium-specific education. However, establishing educational interventions in the nursing home setting is challenging. e-learning is one method of compensating for these difficulties. Therefore, this study aims to develop a curriculum for interdisciplinary e-learning to improve delirium-specific knowledge in HCPs in nursing homes.

**Methods:**

Delirium-specific competencies were formulated on the basis of exploratory setting-independent literature. The competencies were assessed for relevance (very relevant, relevant, less relevant and not relevant) by an expert panel through a two-stage Delphi study that included an integrated workshop. A consensus was assumed if 80% of the experts rated a competence as very relevant or relevant in the first round. Competencies with approval ratings between 75% and 80% after the first round and/or that were critically commented upon were discussed in the subsequent workshop and assessed again in the second round. The competencies that received approval ratings below 75% in the first Delphi round were removed. In the second Delphi round, competencies that did not achieve at least 80% approval were ultimately excluded.

**Results:**

A total of 120 competencies were formulated, including 108 that addressed both disciplines, 4 addressed nurses, and 8 addressed GPs. Nineteen experts participated in the first Delphi round, after which *n*=92 (76.7%) of the competences were approved and *n*=18 (15%) were deleted. A total of 10 (8.3%) of the competencies were critically discussed by 10 experts in the subsequent workshop, of which 6 were deleted. Four competencies (3 addressed nurses and 1 GP) were evaluated by 11 experts in the second Delphi, 3 of which were confirmed (the competence addressing GPs was deleted). Overall, *n*=97 (81.2%) competencies were included in the final curriculum. Of these, *n*=64 (66%) addressed both disciplines, *n*=16 (16.5%) addressed nurses, and *n*=17 (17.5%) addressed GPs.

**Conclusions:**

Delirium is an interdisciplinary challenge. Hence, the majority of our newly developed competencies address both disciplines. However, discipline-specific competencies must be addressed in the development of e-learning. A competence-based curriculum is a necessary basis for providing interdisciplinary e-learning for HCPs in nursing homes.

**Supplementary Information:**

The online version contains supplementary material available at 10.1186/s12909-025-07078-x.

## Background

Delirium is an aetiologically unspecific neuropsychiatric syndrome characterized by an acute onset; a fluctuating course; and impairments in attention, consciousness and cognitive functions [1]. Delirium must be assessed as a nursing and medical emergency, as those affected are at risk of dementia, institutionalism and a higher mortality rate [2]. Nursing home residents are vulnerable to developing delirium due to their advanced age, existing cognitive impairments, high degree of pain issues and the effects of pharmacotherapy [3, 4].

Nurses in nursing homes play a key role in the detection of delirium in residents. Depending on their clinical judgement, the responsible general practitioners (GPs) are notified to validate the diagnosis and initiate further interventions to eliminate the cause of delirium to avoid hospitalization and other adverse events. Although GPs seem to have difficulty recognizing and properly treating delirium [5], nurses appear to have a significant lack of knowledge about delirium, risk factors and assessments [6, 7]. Therefore, interdisciplinary research societies are calling for delirium to gain greater importance and to be better integrated into the training of all health care professionals (HCPs) [8]. In addition, the importance of delirium in nursing homes must be further emphasized, as the number of residents with risk factors for delirium will increase in the future, and distinguishing between delirium and other cognitive impairments remains a major challenge [9].

Although few educational interventions to promote delirium-specific knowledge are available for nurses in nursing homes, no educational interventions could be identified for GPs [10].

Given that the prevention, diagnosis and treatment of delirium must be understood as an interprofessional task and the interaction between nursing staff and GPs in nursing homes is particularly important, educational interventions to increase awareness of delirium should also employ an interprofessional approach [11].

When designing interprofessional training, the different states of knowledge and learning preferences of the target groups must be acknowledged. In Germany, the topic of delirium in nursing home residents does not appear to be structurally anchored in training for nurses, so we assume that nurses have a low level of knowledge about delirium in this population. Delirium tends to be a marginal topic in the further training of GPs. Therefore, we suppose that GPs are aware of delirium but may not know the current state of the art regarding the diagnosis and treatment of delirium. With respect to learning preferences, we assume that professional training for nurses and GPs must be easy and flexible to access, quick to administer and helpful for their clinical practice [12, 13]. These requirements can best be met via asynchronous e-learning. e-learning is the overarching term for the use of information technology and electronic media to support learning activities [14]. Asynchronous learning is particularly suitable in adult education, as learners have the opportunity to access and process information at a time that suits them [15].

In the DeliA project (Delir in Altenpflegeeinrichtungen/Delirium in nursing homes https://osf.io/xkfvh/; funded by the Innovation Committee at the Federal Joint Committee (G-BA) under the number 01 VSF20003; https://delia.info), we aimed to develop interprofessional e-learning to improve the delirium-specific knowledge of nurses and GPs.

The aim of the prevailing study is to develop and obtain a consensus on a competence-based curriculum for e-learning for nurses and GPs to promote delirium-specific knowledge in nursing homes. The competencies should consider discipline-specific learning preferences regarding content.

## Methods

### Research design

A modified Delphi survey was conducted to reach a consensus on the competencies that are the basis of the e-learning curriculum. The Delphi survey is a systematic, multistage evaluation procedure in which experts are asked for their assessment of a topic [16]. Delphi surveys are often used in interdisciplinary health services research and are ideally suited for developing standards, guidelines and curricula for educational interventions to reach a consensus on the content [17]. Our two-stage Delphi survey included two rating rounds and a workshop that occurred between them. The workshop gave the experts the opportunity to enter into a discourse and thus reach a consensus more quickly. The publication is based on the reporting guidelines for Delphi procedures in health sciences [18].

The researchers who conducted the Delphi survey had professional experience in health care/nursing/elderly care (RP, VM, JS) and had participated in graduate studies in nursing (VM, JS), nursing science (RP, VM), health care management (JS), and medicine and public health (HCV). The authors were supported by other members of the DeliA consortium, offering a perspective from the fields of health economics, gerontology, pharmacy and pharmacology.

### Curriculum development

An exploratory literature search was conducted in PubMed and CINAHL to identify existing curricula on delirium and publications about delirium training (VM). Settings other than nursing homes and guidelines from various professional associations were sought. The grey literature was also searched for using Google Scholar. The publications were screened as part of the systematic search for a realist review in the DeliA project [10].

The terms *delirium*, *curriculum*, *guidelines*, *education*, and *knowledge* were linked with Boolean operators*.* The literature included publications in English or German; the search lasted from October 2022 to December 2022. A list of the curricula, guidelines and publications included can be found in Appendix 1.

The competencies were formulated by the first author and reviewed by the interprofessional DeliA team (nursing perspective, medical perspective, and pharmaceutical perspective). The competence concept from the German Qualifications Framework for Lifelong Learning was chosen as the basis for the competencies. This is defined as "the ability and willingness to use knowledge, skills and personal, social and methodological abilities in work or learning situations for professional and personal development” [19]. The competencies developed therefore focused on three key competencies: knowledge, skills and attitudes [20]. A pedagogue was consulted to check and revise the formulation of the competencies.

### Expert panel

We defined experts as individuals with knowledge and experience in a specific subject area; in our study, we defined delirium in nursing home residents [21]. We recruited experts from the following three groups: clinicians with delirium experience from various clinical settings (neurologists, gerontopsychiatrists, geriatricians, anaesthesiologists, intensive care unit nurses, and advanced practice nurses for delirium), clinicians from nursing home settings (geriatric nurses and GPs), and researchers with knowledge of the long-term care setting and/or delirium (health and nursing scientists). All the experts were recruited from the DeliA study group network and first received an informational e-mail, followed by an invitation to an online information session. In this information event, the procedure and the DeliA project were presented in detail.

### First Delphi round

All the experts received the link to an online survey (LimeSurvey) and had the opportunity to look at the informational letter again in detail and make a final decision as to whether they wanted to participate in the Delphi survey. After providing their consent, the experts were asked to rate all competencies developed on a four-point Likert scale (1 = very relevant, 2 = relevant, 3 = less relevant and 4 = not relevant). There are different opinions on how many levels the Likert scale in a Delphi study should have. Often, one finds 4—5 levels [17]. There was also the opportunity to comment on each competence. All the experts were asked to indicate the relevance of the competencies for the two occupational groups, nurses and GPs.

In addition, the experts had to provide sociodemographic and professional biographical data:

- Age: *under 45* years or *45 years and older*,
- Training/studies: *nursing training*, *medicine*, *nursing/health science, other*,
- Specialist nursing training: *intensive care/anaesthesia care, geriatric psychiatric care, palliative care, other,*
- Specialist physician: *anaesthesia/intensive care medicine*, *neurology*, *general practice and family medicine*, *psychiatry*, *other* and
- Work experience in years (median)

The Delphi survey can be found in Appendix 2.The first Delphi round was activated for three weeks, and the experts were reminded every week. After the first survey was completed, the results were evaluated by the research team.

### Workshop

All the experts were invited to an online workshop to discuss the competencies that were not yet classified as part of the consensus or that were commented upon by the experts. For their participation in the workshop, the experts received a financial allowance of 100 euros as an incentive to participate. The competencies that did not reach consensus or that were mentioned in the first Delphi survey round were presented to the workshop participants. Following the discussions, we revised or deleted the competencies. The workshop lasted a total of 2 h.

### Second Delphi round

In the second Delphi round, the experts were asked for a final assessment of the relevance of competencies that were revised in the workshop; ratings were made exclusively using the Likert scale. In the second Delphi round, all the experts provided their sociodemographic and professional biographical data again. The second Delphi round lasted three weeks, and the experts were reminded every week. At the end of the second round, the Delphi survey was completed.

### Data analysis

For the data analysis of the first Delphi round, the results were dichotomized into relevant (1 = very relevant; 2 = relevant) and not relevant (3 = less relevant and 4 = not relevant).

We defined a consensus as 80% relevance [22]. The evaluation scheme was defined a priori in the analysis (Table 1).

**Table 1:** Evaluation scheme for the data analysis of the Delphi study

Cut off value	Commentary	Decision
≥ 80% relevant	No critical comments	Remain unchanged
≥ 80% relevant	Critical comments	Comments are shared in the workshop. Competencies are then discussed, removed or possibly revised and rated again
< 80% and ≥ 75%	No critical comments	Competencies are discussed in the workshop, revised and rated again or removed
< 80% and ≥ 75%	Critical comments	Comments are shared in the workshop. Competencies are discussed in the workshop, possibly revised and rated again or removed
< 75%	No critical comments	Removed
< 75%	Critical comments	Comments are shared in the workshop. Competencies are then discussed, possibly revised and rated again or removed

In the second Delphi round, competencies that did not achieve at least 80% approval were ultimately excluded

The data were analysed descriptively for each competency. We calculated the agreement rate of the whole sample. Additionally, we divided the expert sample into two groups on the basis of their discipline (nursing or medicine) and calculated agreement rates in the two groups. Physicians who also completed training in nursing were considered physicians in the data analysis. Experts who were not trained in nursing or studied human medicine were included in the overall analysis but not in the occupational group-specific analysis. Qualitative comments were summarized narratively. The statistical data analysis was performed using Microsoft Excel, R and RStudio.

## Results

### Curriculum development

On the basis of the results of the literature review, the first author formulated a pool of competencies for nurses, GPs and both professional groups. The competencies were grouped into six modules and encompassed 120 competencies in total:

1. Definitions, complications and consequences
2. Causes and risk factors
3. Symptoms and diagnostics
4. Therapy and management
5. Prevention
6. Terminal delirium

The majority of the initially developed competencies addressed both nurses and GPs, which means that they were identically formulated but addressed a different target group (90%, *n* = 108). Four competencies focused specifically on nurses (3.3%), and 8 (6.7%) focused on GPs.

### Expert characteristics

A total of 30 experts were invited to complete the first Delphi survey. A total of 63.3% (*n* = 19) of the requested experts participated in the first survey, and 36.7% (*n* = 11) of the experts participated in the second Delphi round. The expert characteristics are shown in Table 2.

**Table 2:** Sociodemographic and professional biographical data of the experts

	**First Delphi round**	**Second Delphi round**
Characteristics	Expert panel (*n* = 19)	Expert panel (*n* = 11)
**Age**		
Younger than 45 years	7	5
45 years and older	12	6
**Training/studies**		
Nursing training (EQF: 4)^a^	11	5
Medicine	7	4
Nursing/Health science (at least EQF: 6)^a^	8	4
Other	1	1
**Specialist nursing training**		
Gerontopsychiatric nursing	1	1
Anaesthesia intensive care	1	2
Palliative care	2	0
Other	6	2
**Specialist physician**		
Neurology	3	1
Anaesthesia/Intensive care medicine	3	1
General practice and family medicine	1	2
Psychiatry	1	1
Other	3	0
Work experience (years)	Median: 23	Median: 13

^a^The European Union developed the 8-level European Qualifications Framework (EQF) as a translation tool to make national qualifications easier to understand and more comparable. The level increases according to the level of proficiency; level 1 is the lowest, and level 8 is the highest

The workshop was attended by 10 experts: 5 nurses, 4 physicians and one pharmacist and lasted a total of two hours.

### Descriptive results of the Delphi survey

As a result of the first Delphi round, *n* = 92 from *n* = 120 (76.7%) competencies were approved, including *n* = 44 (36.7%) competencies for nurses and *n* = 48 (40%) competencies for GPs. A total of 18 (15%) of the competencies were removed, of which *n* = 8 (6.7%) were for nurses and *n* = 10 (8.3%) for GPs, as the approval rate was below 75%.

In the workshop, 10 competencies were discussed. Of these, 5 were commented upon, and 5 had an approval rating between 75 and 80%. Of the competencies discussed by the experts, 4 were excluded, 2 were included unchanged in the curriculum, and 4 were revised. Of the excluded competencies, 2 were nurses’ competencies, and 2 were GPs’ competencies.

In the second Delphi round, the revised 4 competencies were evaluated again. Three competencies were ultimately agreed upon after adjustments, and one competency (GP) was removed from the curriculum, as the approval rate was below 80%.

Overall, *n* = 97 (80.8%) competencies were included in the final consented curriculum. Of these, *n* = 64 (66%) addressed both disciplines, *n* = 16 (16.5%) addressed nurses, and *n* = 17 (17.5%) addressed GPs.

#### Module 1 definitions, complications and consequences

Of the 16 competencies (8 competencies for nurses; 8 competencies for GPs), 13 competencies were agreed upon for both disciplines. Six competencies were agreed upon for nurses, and 7 competencies were agreed upon for GPs. The competence *Nurses know figures regarding the prevalence of delirium* was rejected by both disciplines. With respect to the GPs, this competence was rejected by the GPs but not by the nurses; thus, the competence reached a total agreement of ≥ 80%. The competence *Nurses recognize that delirium entails considerable economic costs for the health care system* was rejected by both disciplines. The reference competence for GPs was also removed because the total agreement rate was < 75% (68.42%). However, this competence was rated as relevant by the nurses (80%) but rejected by the physicians (42,86%). The competence *Nurses are aware of possible reasons for large numbers of unreported cases of delirium* was approved because the total agreement was ≥ 80%. Additionally, the nurses rated it as relevant, but the agreement of the physicians was lower*.* No competence was commented on. The results are shown in Table 3.

**Table 3:** Results of the first Delphi round for module 1

**Competencies**	Total *n* = 19	Nurses *n* = 10	Physicians *n* = 7	Decision
Nurses know the definition of delirium	94.74%	100.00%	85.71%	Accepted
General practitioners know the definition of delirium	94.74%	100.00%	85.71%	Accepted
Nurses are aware of the possible complications of delirium	100.00%	100.00%	100.00%	Accepted
General practitioners are aware of the possible complications of delirium	100.00%	100.00%	100.00%	Accepted
**Nurses know figures regarding the prevalence of delirium**	**63.16%**	**60.00%**	**57.14%**	**Removed**
General practitioners know figures regarding the prevalence of delirium	89.47%	100.00%	**71.43%**	Accepted
Nurses are aware of possible reasons for large numbers of unreported cases of delirium	89.47%	100.00%	**71.43%**	Accepted
General practitioners are aware of possible reasons for large numbers of unreported cases of delirium	94.74%	100.00%	85.71%	Accepted
Nurses know why delirium can last from hours to months	84.21%	80.00%	85.71%	Accepted
General practitioners know why delirium can last from hours to months	94.74%	90.00%	100.00%	Accepted
Nurses have a positive attitude towards delirium and are open to further education and training on the subject	100.00%	100.00%	100.00%	Accepted
General practitioners have a positive attitude towards delirium and are open to further education and training on the subject	94.74%	90.00%	100.00%	Accepted
Nurses recognize that delirium is a burden for the affected residents, their relatives and for the interdisciplinary team	100.00%	100.00%	100.00%	Accepted
General practitioners recognize that delirium is a burden for the affected residents, their relatives and for the interdisciplinary team	100.00%	100.00%	100.00%	Accepted
**Nurses recognize that delirium entails considerable economic costs for the health care system**	**52.63%**	**50.00%**	**42.86%**	**Removed**
**General practitioners recognize that delirium entails considerable economic costs for the health care system**	**68.42%**	80.00%	**42.86%**	**Removed**

#### Module 2 causes and risk factors

In module 2, out of 16 competencies (8 competencies for nurses; 8 competencies for GPs), 5 competencies for nurses and 7 competencies for GPs were agreed upon. Three nurses’ competencies were rejected by the nurses and physicians: *Nurses describe possible pathophysiological mechanisms for the development of delirium*, *Nurses describe relevant theories on the pathophysiology of delirium* and *Nurses initiate case discussions to assess the risk of residents developing delirium.* The competence *General practitioners initiate case discussions to assess the risk of residents developing delirium* was also rejected (73.68%)*.* The physicians considered this to be relevant (85.71%), but the nurses did not (60%). The competence *General practitioners describe relevant theories on the pathophysiology of delirium* was included in the curriculum, as the overall approval rate was above 80%, although the physicians rated competence as significantly less relevant (57.14%) than the nurses did (100%). The competence *Nurses work collaboratively with other colleagues and across professions* and the referring competence for GPs were both rejected by the physicians (71.43%) but approved by the nurses (100%). The competencies were ultimately included, as the total agreement was ≥ 80%. No critical comments were made. The results of module 2 of the first Delphi round are presented in Table 4.

**Table 4:** Results of the first Delphi round for module 2

**Competencies**	Total *n* = 19	Nurses *n* = 10	Physicians *n* = 7	Decision
**Nurses describe possible pathophysiological mechanisms for the development of delirium**	**36.84%**	**50.00%**	**14.29%**	**Removed**
General practitioners describe possible pathophysiological mechanisms for the development of delirium	94.74%	100.00%	85.71%	Accepted
**Nurses describe relevant theories on the pathophysiology of delirium**	**26.32%**	**40.00%**	**0.00%**	**Removed**
General practitioners describe relevant theories on the pathophysiology of delirium	84.21%	100.00%	**57.14%**	Accepted
Nurses describe predisposing risk factors for the development of delirium	100.00%	100.00%	100.00%	Accepted
General practitioners describe predisposing risk factors for the development of delirium	100.00%	100.00%	100.00%	Accepted
Nurses describe precipitating factors for the development of delirium	100.00%	100.00%	100.00%	Accepted
General practitioners describe precipitating factors for the development of delirium	100.00%	100.00%	100.00%	Accepted
Nurses identify and communicate existing risk profiles and triggering factors for delirium in residents	94.74%	90.00%	100.00%	Accepted
General practitioners identify and communicate existing risk profiles and triggering factors for delirium in residents	89.47%	80.00%	100.00%	Accepted
**Nurses initiate case discussions to assess the risk of residents developing delirium**	**73.68%**	**70.00%**	**71.43%**	**Removed**
**General practitioners initiate case discussions to assess the risk of residents developing delirium**	**73.68%**	**60.00%**	85.71%	**Removed**
Nurses recognize that delirium is a multifactorial phenomenon	94.74%	100.00%	85.71%	Accepted
General practitioners recognize that delirium is a multifactorial phenomenon	94.74%	100.00%	85.71%	Accepted
Nurses work collaboratively with other colleagues and across professions	89.47%	100.00%	**71.43%**	Accepted
General practitioners work collaboratively with other colleagues and across professions	89.47%	100.00%	**71.43%**	Accepted

#### Module 3 symptoms and diagnostics

In module 3 (9 competencies for nurses; 11 competencies for GPs), 18 out of 20 competencies were agreed upon, including 7 competencies for nurses and all competencies for GPs. The competence of *General practitioners diagnose delirium according to the standard diagnostic criteria* was assessed as less relevant by the physicians (71.43%) than by the nurses (100%) but was nevertheless included in the curriculum because the overall agreement was ≥ 80%. Two competencies were agreed upon by the nurses but rejected by the physicians and received comments: *Nurses explain why distinguishing among delirium, dementia and depression is a challenge* and *Nurses differentiate between subtypes of delirium.* Regarding the first competence, a rewording was recommended in the comment; regarding the second competence, in the comment, it was stated that the subtypes are less relevant. These competencies were discussed in the subsequent workshop. More detailed information on the results of the first Delphi survey can be found in Table 5.

**Table 5:** Results of the first Delphi round for module 3

**Competencies**	Total *n* = 19	Nurses *n* = 10	Physicians *n* = 7	Decision
Nurses know valid screening instruments for assessing delirium	94.74%	100.00%	85.71%	Accepted
Nurses feel confident in their ability to screen for delirium	94.74%	100.00%	85.71%	Accepted
General practitioners know valid diagnostic tools	100.00%	100.00%	100.00%	Accepted
General practitioners diagnose delirium according to the standard diagnostic criteria	89.47%	100.00%	**71.43%**	Accepted
General practitioners differentiate between various forms of delirium, alcohol withdrawal delirium and other acute neuropsychiatric conditions	100.00%	100.00%	100.00%	Accepted
General practitioners feel confident in their ability to diagnose delirium	94.74%	100.00%	85.71%	Accepted
Nurses describe symptoms of delirium	100.00%	100.00%	100.00%	Accepted
General practitioners describe symptoms of delirium	94.74%	90.00%	100.00%	Accepted
**Nurses explain why distinguishing among delirium, dementia and depression is a challenge**	**73.68%**	100.00%	**28.57%**	**Discussed, adapted and rated again**
General practitioners explain why distinguishing among delirium, dementia and depression is a challenge	94.74%	100.00%	85.71%	Accepted
**Nurses differentiate between subtypes of delirium**	**78.95%**	100.00%	**42.86%**	**Discussed, adapted and rated again**
General practitioners differentiate between subtypes of delirium	94.74%	100.00%	85.71%	Accepted
Nurses identify possible causes of delirium in residents	89.47%	90.00%	85.71%	Accepted
General practitioners identify possible causes of delirium in residents	89.47%	90.00%	85.71%	Accepted
Nurses use screening or diagnostic instruments safely and correctly	100.00%	100.00%	100.00%	Accepted
General practitioners use screening or diagnostic instruments safely and correctly	94.74%	90.00%	100.00%	Accepted
Nurses provide immediate feedback to the multidisciplinary team if delirium is suspected	100.00%	100.00%	100.00%	Accepted
General practitioners provide immediate feedback to the multidisciplinary team if delirium is suspected	100.00%	100.00%	100.00%	Accepted
Nurses recognize delirium as an emergency situation that requires immediate intervention	100.00%	100.00%	100.00%	Accepted
General practitioners recognize delirium as an emergency situation that requires immediate intervention	100.00%	100.00%	100.00%	Accepted

With respect to competence, *Nurses explain why distinguishing among delirium, dementia and depression is a challenge* receive high approval from the experts at the workshop. A total of four experts explained in the discussion that the ability of nurses to differentiate among delirium, dementia and depression is very important. The formulation of the competence was criticized, as the focus should not be on the clear distinction between the syndromes and clinical pictures, but rather on the essential distinguishing features. We revised the competence as follows: *Nurses differentiate among delirium, dementia and depression*. In the second Delphi round, the revised competence yielded an agreement of 100% and was therefore left in the curriculum. Regarding the competence *Nurses differentiate among subtypes of delirium,* the experts discussed that it is essential to know the different subtypes of delirium. Finally, we did not revise the competence, and it proceeded to the second Delphi round. It yielded an agreement of 90.91% and was therefore left in the curriculum.

#### Module 4 therapy and management

In module 4, out of a total of 25 competencies (12 competencies for nurses; 13 competencies for GPs), 8 were agreed upon for nurses, and 10 were agreed upon for GPs. Four competencies were removed because the agreement was < 80%: The competence *Nurses know the key aspects of liability law when dealing with residents with delirium* was accepted by nurses (90%) but rejected by physicians (42.86%). This resulted in an overall approval rating of 73.68%, meaning that the competence was excluded. The competence *Nurses weigh up the use of pharmacological measures together with the interprofessional team with regard to potential side effects* was also rejected (57.89%). This competence was also approved by the nurses (80%), whereas it was clearly rejected by the doctors (28.57%). Although the competence *General practitioners inform and train relatives of affected residents about delirium and accompany them* was rejected by both nurses (40%) and physicians (42.86%) the competence *General practitioners closely accompany and monitor residents with delirium* was approved by the physicians (85.71%) but finally rejected by the nurses (50%). In addition, the competence *Nurses feel competent to treat delirium* was rated as less relevant by physicians (71.43%) than by nurses (100%). In the end, however, the competence was included in the curriculum because of an overall agreement of ≥ 80%.

Three competencies were commented on*: Nurses name possible medications that can be given in the acute phase of delirium*, *Nurses see relatives as an important component in the treatment of delirium;* and *General practitioners see relatives as an important component in the treatment of delirium.* With respect to the first competence, rewording was recommended; however, the agreement rate was low (52.63%). Regarding the two other competencies, the comments emphasized that relatives are not always a resource. The three competencies were discussed in the workshop. The results are presented in Table 6.

**Table 6:** Results of the first Delphi round for module 4

**Competencies**	Total *n* = 19	Nurses *n* = 10	Physicians *n* = 7	Decision
**Nurses name possible medications that can be given in the acute phase of delirium**	**52.63%**	**70.00%**	**28.57%**	**Discussed and removed**
Nurses request medical assistance if delirium is suspected	94.74%	100.00%	85.71%	Accepted
General practitioners know possible medications and side effects and the dosages that can be given in the acute phase of delirium	100.00%	100.00%	100.00%	Accepted
General practitioners know possible medications, their side effects, and the dosages that can be given in the acute phase of delirium	100.00%	100.00%	100.00%	Accepted
General practitioners demand support from nursing staff in the treatment of existing delirium	100.00%	100.00%	100.00%	Accepted
Nurses describe nonpharmacological measures for the treatment of delirium	94.74%	100.00%	85.71%	Accepted
General practitioners describe nonpharmacological measures for the treatment of delirium	84.21%	**70.00%**	100.00%	Accepted
**Nurses know the key aspects of liability law when dealing with residents with delirium**	**73.68%**	90.00%	**42.86%**	**Removed**
General practitioners know the key aspects of liability law when dealing with residents with delirium	89.47%	100.00%	**71.43%**	Accepted
Nurses identify and eliminate possible causes of delirium	100.00%	100.00%	100.00%	Accepted
General practitioners identify and eliminate possible causes of delirium	100.00%	100.00%	100.00%	Accepted
Nurses closely accompany and monitor residents with delirium	100.00%	100.00%	100.00%	Accepted
**General practitioners closely accompany and monitor residents with delirium**	**68.42%**	**50.00%**	85.71%	**Removed**
Nurses inform and train relatives of affected residents about delirium and accompany them	100.00%	100.00%	100.00%	Accepted
**General practitioners inform and train relatives of affected residents about delirium and accompany them**	**47.37%**	**40.00%**	**42.86%**	**Removed**
Nurses communicate empathically and sensitively with residents with delirium	100.00%	100.00%	100.00%	Accepted
General practitioners communicate empathically and sensitively with residents with delirium	94.74%	90.00%	100.00%	Accepted
Nurses develop an interprofessional, facility-specific pathway for the treatment of delirium	89.47%	90.00%	85.71%	Accepted
General practitioners develop an interprofessional, facility-specific pathway for the treatment of delirium	94.74%	90.00%	100.00%	Accepted
**Nurses weigh up the use of pharmacological measures together with the interprofessional team with regard to potential side effects**	**57.89%**	80.00%	**28.57%**	**Removed**
General practitioners weigh up the use of pharmacological measures together with the interprofessional team with regard to potential side effects	94.74%	90.00%	100.00%	Accepted
Nurses feel competent to treat delirium	84.21%	90.00%	**71.43%**	Accepted
General practitioners feel competent to treat delirium	100.00%	100.00%	100.00%	Accepted
**Nurses see relatives as an important component in the treatment of delirium**	100.00%	100.00%	100.00%	**Discussed and adopted unchanged**
**General practitioners see relatives as an important component in the treatment of delirium**	89.47%	90.00%	85.71%	**Discussed and adopted unchanged**

Regarding the competence *Nurses name possible medications that can be given in the acute phase of delirium,* a professional group-specific discrepancy was observed in the discussion of this competence. Although one nursing professional spoke out in favour of identifying the medication, the physicians and one pharmacist saw no need for this competence and were therefore in favour of removing the competence. The nursing staff noted that the wording would definitely need to be adapted if the competence were to be retained. In addition, it was important for the nursing staff to classify competence so that it was clear that the medication only served to combat symptoms and was the last resort. The pharmacist noted that background knowledge of the medication was essential for its administration. At the end of the discussion, it was agreed that the competence should be removed.

Subsequently, the competence *Nurses see relatives as an important component in the treatment of delirium* and the similar competence for GPs were discussed. Work with relatives was generally considered very important and beneficial by the experts. However, how relatives can be specifically involved in the recognition and/or treatment of delirium depends very much on the situation and must be assessed individually by the responsible care professional. Finally, the competencies adopted were unchanged in the curriculum, consistent with the discussion.

#### Module 5 prevention

In Module 5, out of 20 competencies (10 competencies for nurses; 10 competencies for GPs), 9 competencies for nurses and 3 competencies for GPs were agreed upon. Four competencies were removed because they were rejected by both the nurses and the physicians*: General practitioners use nonpharmacological measures to prevent delirium, General practitioners use measures for cognitive stimulation and emotional relief, General practitioners use reorienting measures to shape the environment*, *General practitioners promote regular contact between residents and their relatives,* and *General practitioners ensure that residents at high risk of delirium are cared for by people they trust.* The competence *Nurses initiate a medication review at regular intervals* was rejected by physicians (71.43%), whereas nurses clearly agreed with it (90%). The opposite was the case for the competence *Nurses recognize the importance of communication between nurses and physicians with respect to the prescription of sedatives/neuroleptics*, where nurses rated competence as less relevant (70%) than physicians did (100%). Overall, however, both competencies were included in the curriculum, as they received an overall approval rating of > 80%.

Two competencies *Nurses are sensitized to the fact that prevention is of great importance both individually and economically* (78.95%) and *General practitioners are sensitized to the fact that prevention is of great importance both individually and economically* (78.95%) had approval ratings between 75 and 80%, so these were discussed in the workshop.

Two competencies were commented on *General practitioners use nonpharmacological measures to prevent delirium* and *General practitioners initiate case discussions and team discussions with residents at risk of delirium.* Regarding the first competency, it was noted that interprofessional consultation is necessary to prevent delirium. Regarding the second competency, it was reported that the role is unclear. However, the agreement rate of both competencies was < 80%. The results of the first Delphi survey are presented in Table 7.

**Table 7:** Results of the first Delphi round for module 5

**Competencies**	Total *n* = 19	Nurses *n* = 10	Physicians *n* = 7	Decision
Nurses use nonpharmacological measures to prevent delirium	100.00%	100.00%	100.00%	Accepted
**General practitioners use nonpharmacological measures to prevent delirium**	**57.89%**	**50.00%**	**57.14%**	**Discussed and removed**
Nurses use measures for cognitive stimulation and emotional relief	94.74%	100.00%	85.71%	Accepted
**General practitioners use measures for cognitive stimulation and emotional relief**	**31.58%**	**20.00%**	**28.57%**	**Removed**
Nurses use reorienting measures to shape the environment	100.00%	100.00%	100.00%	Accepted
**General practitioners use reorienting measures to shape the environment**	**47.37%**	**30.00%**	**57.14%**	**Removed**
Nurses initiate a medication review at regular intervals	84.21%	90.00%	**71.43%**	Accepted
General practitioners initiate a medication review at regular intervals	100.00%	100.00%	100.00%	Accepted
Nurses promote regular contact between residents and their relatives	94.74%	90.00%	85.71%	Accepted
**General practitioners promote regular contact between residents and their relatives**	**31.58%**	**20.00%**	**28.57%**	**Removed**
Nurses communicate actively and openly with residents	100.00%	100.00%	100.00%	Accepted
General practitioners communicate actively and openly with residents	94.74%	100.00%	85.71%	Accepted
Nurses initiate case discussions and team discussions on residents at risk of delirium	94.74%	90.00%	100.00%	Accepted
**General practitioners initiate case discussions and team discussions on residents at risk of delirium**	**78.95%**	**70.00%**	85.71%	**Discussed adapted, rated again and removed**
Nurses ensure that residents at high risk of delirium are cared for by people they trust	89.47%	90.00%	85.71%	Accepted
**General practitioners ensure that residents at high risk of delirium are cared for by people they trust**	**42.11%**	**10.00%**	**71.43%**	**Removed**
**Nurses are sensitized to the fact that prevention is of great importance both individually and economically**	**78.95%**	80.00%	**71.43%**	**Discussed and removed**
**General practitioners are sensitized to the fact that prevention is of great importance both individually and economically**	**78.95%**	80.00%	**71.43%**	**Discussed and removed**
Nurses recognize the importance of communication between nurses and physicians with regard to the prescription of sedatives/neuroleptics	84.21%	**70.00%**	100.00%	Accepted
General practitioners recognize the importance of communication between nurses and physicians with regard to the prescription of sedatives/neuroleptics	94.74%	90.00%	100.00%	Accepted

With respect to the competence *General practitioners use nonpharmacological measures to prevent*, all workshop participants agreed that non-pharmacological measures were the responsibility of the nursing staff and that nurses were allowed to initiate and perform them independently. Interdisciplinary consultation on nonpharmacological delirium prevention is not considered relevant. Against this background, it was decided to remove the competence from the curriculum.

With respect to the competence *General practitioners initiate case discussions and team discussions on residents at risk of delirium*, the experts did not consider competence to be feasible. GPs would not have the resources to initiate case discussions, and would not fit into the existing care structures. It was therefore decided to adapt the competence and rate it again in the second Delphi round. In this context, the adapted competence *General practitioners initiate case discussions with the team to raise awareness of delirium* was not agreed upon (54.5%). Consequently, this competence was removed.

Regarding the two competencies *Nurses/General practitioners are sensitized to the fact that prevention is of great importance both individually and economically*, it was discussed that economic effects of delirium prevention were considered important by the experts from all professional groups but were simultaneously considered negligible for the curriculum. Thus, the economic aspect always plays a subordinate role but is not central with respect to the curriculum content. Both competencies were removed.

#### Module 6 terminal delirium

In module 6, 19 of the 23 competencies (11 competencies for nurses; 12 competencies for GPs) were agreed upon for nurses, and 10 were agreed upon for GPs. Both nurses and physicians did not ascribe high relevance to the competence *General practitioners ask about the concerns, problems and needs of the relatives of dying residents in order to address these adequately and sensitively* (68.42%), so it was removed. *Nurses know of drugs that can be used to treat terminal delirium with a heavy symptom burden* was rejected because of a lack of approval (68.42%). The competency was approved by 80% of the nurses but a minority of the physicians (42.86%). The competence *General practitioners promote a calm and reorienting environment for residents with terminal delirium* was removed, as the overall relevance rating was 68.42%. This competence was rejected by 50% of the nurses but agreed upon by 85.71% of the physicians*.* The two competencies *General practitioners provide appropriate care and training for relatives and see them as an important component in alleviating the symptoms of delirium in dying residents* and *Nurses take a critical stance on the regular administration of psychotropic drugs for terminal delirium* were rated as relevant by 70% of the nurses, whereas the physicians agreed with the competencies 100% in each case. In the end, the overall approval rate for both competencies was 84.21%; thus, both competencies were included in the final curriculum*.*

The competence *Nurses distinguish a terminal delirium, from a delirium that arises due to an underlying physical cause and must be considered a potential emergency* was discussed in the workshop because the wording must be adjusted. The results of module 6 are reported in Table 8.

**Table 8:** Results of the first Delphi round for module 6

**Competencies**	Total *n* = 19	Nurses *n* = 10	Physicians *n* = 7	Decision
General practitioners prescribe on-demand medication to reduce the symptom burden in terminal delirium	100.00%	100.00%	100.00%	Accepted
Nurses distinguish a terminal delirium, from a delirium that arises due to an underlying physical cause and must be considered a potential emergency	**78.95%**	90.00%	**71.43%**	**Discussed adapted and rated again**
General practitioners distinguish a terminal delirium, from a delirium that arises due to an underlying physical cause and must be considered a potential emergency	94.74%	100.00%	85.71%	Accepted
**Nurses know of drugs that can be used to treat terminal delirium with a heavy symptom burden**	**68.42%**	80.00%	**42.86%**	**Removed**
General practitioners know of drugs that can be used to treat terminal delirium with a heavy symptom burden	100.00%	100.00%	100.00%	Accepted
Nurses are familiar with specific symptoms such as positive hallucinations in terminal delirium	100.00%	100.00%	100.00%	Accepted
General practitioners are familiar with specific symptoms such as positive hallucinations in terminal delirium	100.00%	100.00%	100.00%	Accepted
Nurses promote a calm and reorienting environment for residents with terminal delirium	100.00%	100.00%	100.00%	Accepted
**General practitioners promote a calm and reorienting environment for residents with terminal delirium**	**68.42%**	**50.00%**	85.71%	**Removed**
Nurses communicate calmly and empathetically with dying residents and ensure continuous care	100.00%	100.00%	100.00%	Accepted
General practitioners communicate calmly and empathetically with dying residents and ensure continuous care	94.74%	100.00%	85.71%	Accepted
Nurses provide appropriate care and training for relatives and see them as an important component in alleviating the symptoms of delirium in dying residents	100.00%	100.00%	100.00%	Accepted
General practitioners provide appropriate care and training for relatives and see them as an important component in alleviating the symptoms of delirium in dying residents	84.21%	**70.00%**	100.00%	Accepted
Nurses ask about the concerns, problems and needs of the relatives of dying residents in order to address these adequately and sensitively	94.74%	90.00%	100.00%	Accepted
**General practitioners ask about the concerns, problems and needs of the relatives of dying residents in order to address these adequately and sensitively**	**68.42%**	**60.00%**	**71.43%**	**Removed**
Nurses argue that the intensity of interventions should be guided by the dying resident’s subjective level of suffering and possible danger to themselves and others	100.00%	100.00%	100.00%	Accepted
General practitioners argue that the intensity of interventions should be guided by the dying resident’s subjective level of suffering and possible danger to themselves and others of the dying resident	94.74%	90.00%	100.00%	Accepted
Nurses are critical of unnecessary hospital admissions for dying residents	100.00%	100.00%	100.00%	Accepted
General practitioners are critical of unnecessary hospital admissions for dying residents	100.00%	100.00%	100.00%	Accepted
Nurses take a critical stance on the regular administration of psychotropic drugs for terminal delirium	84.21%	**70.00%**	100.00%	Accepted
General practitioners take a critical stance on the regular administration of psychotropic drugs for terminal delirium	94.74%	90.00%	100.00%	Accepted
Nurses see relatives as an important component in alleviating the symptoms of delirium in dying residents	100.00%	100.00%	100.00%	Accepted
General practitioners see relatives as an important component in alleviating the symptoms of delirium in dying residents	100.00%	100.00%	100.00%	Accepted

With respect to the competence *Nurses distinguish terminal delirium from delirium that arises due to an underlying physical cause and must be considered a potential emergency*, it was explained that the distinction is very complex and that even physicians have problems with it. It was therefore decided to reformulate this competency to emphasize knowledge instead of the clear distinction and to ask about it again. We revised the competency as follows: *Nurses know that delirium can be part of the terminal phase of life and is not a potential emergency*. In the second Delphi round, the revised competency yielded 100% agreement.

## Discussion

In this modified Delphi study, a competence-based curriculum for e-learning to promote delirium-specific knowledge of HCPs in nursing homes was consented by a panel of experts. This is the first known study from Germany to address the phenomenon of delirium in nursing homes and to address the lack of knowledge of HCPs in a structured manner. The interdisciplinary curriculum was designed in such a way that the majority of the competencies it contains were formulated for both nurses and physicians. Even after completion of the Delphi survey, 66.6% of the competencies (n = 64) of the final 97 agreed-upon competencies were included in the curriculum for both professional groups. The majority of the competencies were considered relevant across disciplines, which confirms the interprofessional concept of e-learning. Nevertheless, there are focal points in terms of content, as 16.5% of competencies (n = 16) were agreed upon, specifically for nurses, and 17.6% (n = 17) were agreed upon for GPs. The analyses also demonstrate that the assessment of the relevance of some competencies differs within occupational groups. This led to clarification, as some competencies were only classified as relevant for one occupational group and included in the curriculum. However, this also often led to competencies being removed because the other professional group rejected the competence. Alternatively, it led to competencies being retained even though their own professional group rejected the competence.

Although no interdisciplinary curriculum could be found in the literature search, a curriculum was identified that contains delirium-specific content that should be taught as part of medical studies for students [23]. This curriculum was divided into three main areas: *Aetiology, epidemiology and pathophysiology*; *Diagnostics;* and *Management*. In terms of content, there are only a few differences between this curriculum and our version for nursing homes with respect to GPs. One difference is that we also specifically address delirium in the dying phase. The curriculum for medical students has also been broader and focused on different clinical settings [23]. In parallel, delirium prevention tends not to be seen as a medical task. In our curriculum, most of the competencies for GPs were rejected in the prevention module, whereas almost all of them were approved for nurses. In addition, the curriculum by Copeland et al. mentions that it is important to know that delirium can be prevented [23]. Furthermore, Copeland's curriculum states specific theories on pathophysiology, whereas our curriculum provides only a brief excursus on the subject. The fact that delirium accelerates cognitive decline is also mentioned in our version but is not discussed in detail. The same applies to the problem that some HCPs have a negative attitude towards patients with delirium. However, both curricula clearly focus on the ability of physicians to diagnose and treat delirium. The fact that the management and treatment of delirium is regarded as a core task of medical professionals in nursing homes can also be confirmed elsewhere [24].

Although the DeliA workshop emphasized that it is not necessary for nursing staff to know the pathophysiological mechanisms and medications used to treat severe symptoms of existing delirium, nursing staff in nursing homes play a leading role in the prevention of delirium.

Special delirium prevention programs for nursing homes should therefore be developed and implemented in the direction of nursing staff.

As a result of the Delphi study, the differentiation among dementia, delirium and delirium superimposed on dementia must be assessed as an elementary competence in nursing homes. The quality of care for the diagnosis and treatment of delirium in nursing homes is based on this competence. Nurses in particular should therefore be sensitized to differentiate among the essential core elements of dementia, depression and delirium. In another study, the extent to which delirium plays a role in Scottish nursing degree courses was investigated [25]. Here, there were discrepancies in content, as the differentiation between dementia and delirium was not correctly considered. This makes it clear that this difference cannot be taught correctly even in the context of nursing training.

In summary, nursing staff are not expected to be able to explain the pathophysiology of delirium, name medications or weigh the side effects. GPs rarely have the opportunity for the prevention of delirium or nonpharmacological measures. These results clearly show the responsibilities in delirium management and could improve cooperation if the competencies of other professional groups are clear.

The curriculum can serve as a basis for the development of standards and procedural instructions for dealing with delirium in nursing homes, which are aimed at both nursing staff and general practitioners. This enables a clear definition of roles and responsibilities with the aim of sustainably improving the quality of care for residents. The results of the Delphi study illustrate the current care situation in nursing homes, whereby a central dilemma manifests itself: GPs are not usually on site at all times, and delirium must be considered an acute emergency situation that requires rapid and effective intervention. Against this background, advanced nursing skills, e.g. in the diagnosis and treatment of delirium, could be of considerable importance. In particular, extended nursing roles and tasks, as already implemented by advanced practice nurses in hospitals [26, 27], could have a significant impact on the quality of delirium care in nursing homes and also help to relieve the burden on GPs. Pilot projects and further research on the transfer of such nursing roles from the hospital to the nursing home are needed to fully assess the potential and practical implementation of such innovative approaches to delirium in nursing homes.

### Strengths and limitations

The particular strength of the e-learning curriculum is explained by the interprofessional approach to raising awareness of delirium in nursing homes. Notably, in Germany, nursing staff in nursing homes work autonomously, and GPs visit residents at regular intervals or on an on call basis. The curriculum developed now makes it clear which steps in the process should be undertaken by professional groups in the context of delirium care.

Nevertheless, the limitations of this Delphi study must be noted. Our approach differs from a classic Delphi version in that we integrated a workshop between the two rounds, which removed the anonymity of the experts who participated in the workshop. The workshop was very enriching for the knowledge process but had the major bias that dominant personalities were given more consideration in the discussion process than were reserved experts. An attempt was made to mitigate this through neutral moderation by the authors. Another limitation is that we conducted only two rating rounds due to limited resources. However, it should be noted that a consensus on the majority of the competencies was already reached after the first Delphi round.

Only national experts were involved in the Delphi study; thus, the results may only be transferable to other health care systems to a limited extent. International expertise was therefore not included in the curriculum consensus. However, this can also be seen as positive feature, as delirium does not appear to be perceived as a phenomenon in nursing homes in Germany.

Another limitation is the small number of participants. Given that the topic of delirium in nursing homes is not yet well known in Germany and possible uncertainties arise in this context, this could explain the lower response rate of the experts, which was 63.3% in the first Delphi round, compared with 96% in the study by Copeland et al. [23]. In addition, the participation rate in the second Delphi round was even lower; however, this is a well-known phenomenon. The low participation rate may bias the results with respect to agreement or disagreement due to an unequal proportion of participants with a nursing or medical background.

Another limitation is that the curriculum was only partially developed from scratch together by geriatric nurses and GPs. They were involved only in the form of an expert committee when the first draft of the curriculum was available and were possibly influenced by it. Given that the topic of delirium has not yet been researched to any great extent in the area of nursing homes, finding experts who are familiar with the condition and have practical experience in nursing homes is also challenging.

## Conclusion

Delirium is a serious emergency and an interdisciplinary challenge in nursing homes. The majority of our newly developed competencies for interdisciplinary e-learning address both disciplines, nursing and medicine. Hence, discipline-specific competencies must be addressed, and different learning needs must be acknowledged in the development of an e-learning curriculum. The disagreement about which professional group should have which skills in delirium management/prevention could also be expressed through interprofessional collaboration and have a negative impact on it, as there is an expectation that the other professional group may not fulfil the necessary requirements. This makes interprofessional training courses and team measures in which this is addressed all the more important. The consented competence-based curriculum will serve as a basis for developing e-learning to increase knowledge about delirium and potentially improve the quality of care for residents in nursing homes. On the basis of the results of the Delphi study, we developed e-learning content and commissioned an agency to jointly implement the digital content in a learning management system. The final e-learning will be usable for health care professionals free of charge and is accessible via the project website (https://delia.info).

## Supplementary Information

Supplementary Material 1.

Supplementary Material 2.

## Data Availability

The datasets generated and analysed during the current study are available upon reasonable request from the corresponding author.

## Abbreviations

GP: General Practitioners
HCP: Health Care Professionals
