# Supplementary material for: Development of a curriculum for interdisciplinary e-learning on delirium in nursing homes—a modified Delphi study
Source: BMC Med Educ. 2025 Apr 7;25:493. doi: 10.1186/s12909-025-07078-x (PMC11977890; doi:10.1186/s12909-025-07078-x)
Supplement: Supplementary file 1 — Supplementary Material 1. [file 12909_2025_7078_MOESM1_ESM.docx]

**Appendix 1 Included publications**

Copeland, C., Fisher, J., & Teodorczuk, A. (2018). Development of an international undergraduate curriculum for delirium using a modified delphi process. *Age Ageing*, *47*(1), 131-137. <https://doi.org/10.1093/ageing/afx133>

Leitlinienprogramm Onkologie (Deutsche Krebsgesellschaft, Deutsche Krebshilfe, AWMF): Palliativmedizin für Patienten mit einer nicht heilbaren Krebserkrankung, Kurzversion 2.3 , 2021, AWMF-Registernummer: 128/001OL, Retrieved 12.04.2023 https://www.leitlinienprogramm-onkologie.de/leitlinien/palliativmedizin/

NHS Greater Manchester (2019): A Greater Manchester Approach To Delirium. Retrieved 12.04.2023, Available from [A-GM-Approach-to-Delirium-Jun2019.pdf (dementia-united.org.uk)](https://dementia-united.org.uk/wp-content/uploads/sites/4/2019/10/A-GM-Approach-to-Delirium-Jun2019.pdf)

NHS Northern England (2018): Delirium Curriculum for Acute Hospital staff. Retrieved 12.04.2023, Available from [Delirium-curriculum-for-acute-hospital-staff-v4.0-final.pdf (england.nhs.uk)](https://www.england.nhs.uk/north/wp-content/uploads/sites/5/2018/12/Delirium-curriculum-for-acute-hospital-staff-v4.0-final.pdf)

National Institute for Health and Care Excellence (2010): Delirium: prevention, diagnosis and management in hospital and long-term care. Last updated: 18. January 2023. Retrieved 12.04.2023, Available from [Delirium: prevention, diagnosis and management in hospital and long-term care (nice.org.uk)](https://www.nice.org.uk/guidance/cg103/resources/delirium-prevention-diagnosis-and-management-in-hospital-and-longterm-care-pdf-35109327290821)

Scottish Intercollegiate Guidelines Network (SIGN). Risk reduction and management of delirium. Edinburgh: SIGN; 2019. (SIGN publication no. 157). [March 2019]. Retried 12.04.2023, URL: http://www.sign.ac.uk
