# Supplementary material for: Development of a curriculum for interdisciplinary e-learning on delirium in nursing homes—a modified Delphi study
Source: BMC Med Educ. 2025 Apr 7;25:493. doi: 10.1186/s12909-025-07078-x (PMC11977890; doi:10.1186/s12909-025-07078-x)
Supplement: Supplementary file 2 — Supplementary Material 2. [file 12909_2025_7078_MOESM2_ESM.docx]

**Delphi survey**

| **Characteristics** | **Responses** |
| --- | --- |
| How old are you? | Younger than 45 years O 45 years and older O |
| What kind of training/studies do you have? | Nursing training O Medicine O Nursing/Health Science O Other O |
| What kind of specialised nursing training did you complete? | Gerontopsychiatric care O Anaesthesia/Intensive care O Palliative care O Other O |
| What kind of specialist physician did you complete? | Neurology O Anaesthesia/intensive care O General practice and family medicine O Psychiatry O Other O |
| How many years of work experience do you have? | (in years) |

Module 1

| **Competencies** | **Relevance** | **Comment** |
| --- | --- | --- |
| Nurses know the definition of delirium. | Very relevant O relevant O less relevant O not relevant O |  |
| General practitioners know the definition of delirium. | Very relevant O relevant O less relevant O not relevant O |  |
| Nurses are aware of the possible complications of delirium. | Very relevant O relevant O less relevant O not relevant O |  |
| General practitioners are aware of the possible complications of delirium. | Very relevant O relevant O less relevant O not relevant O |  |
| Nurses know figures regarding the prevalence of delirium. | Very relevant O relevant O less relevant O not relevant O |  |
| General practitioners know figures regarding the prevalence of delirium. | Very relevant O relevant O less relevant O not relevant O |  |
| Nurses are aware of possible reasons for large numbers of unreported cases of delirium. | Very relevant O relevant O less relevant O not relevant O |  |
| General practitioners are aware of possible reasons for large numbers of unreported cases of delirium. | Very relevant O relevant O less relevant O not relevant O |  |
| Nurses know why delirium can last from hours to months. | Very relevant O relevant O less relevant O not relevant O |  |
| General practitioners know why delirium can last from hours to months. | Very relevant O relevant O less relevant O not relevant O |  |
| Nurses have a positive attitude towards delirium and are open to further education and training on the subject. | Very relevant O relevant O less relevant O not relevant O |  |
| General practitioners have a positive attitude towards delirium and are open to further education and training on the subject. | Very relevant O relevant O less relevant O not relevant O |  |
| Nurses recognize that delirium is a burden for the affected residents, their relatives and for the interdisciplinary team. | Very relevant O relevant O less relevant O not relevant O |  |
| General practitioners recognize that delirium is a burden for the affected residents, their relatives and for the interdisciplinary team. | Very relevant O relevant O less relevant O not relevant O |  |
| Nurses recognize that delirium entails considerable economic costs for the health care system. | Very relevant O relevant O less relevant O not relevant O |  |
| General practitioners recognize that delirium entails considerable economic costs for the health care system. | Very relevant O relevant O less relevant O not relevant O |  |

Module 2

| **Competencies** | **Relevance** | **Comment** |
| --- | --- | --- |
| Nurses describe possible pathophysiological mechanisms for the development of delirium. | Very relevant O relevant O less relevant O not relevant O |  |
| General practitioners describe possible pathophysiological mechanisms for the development of delirium. | Very relevant O relevant O less relevant O not relevant O |  |
| Nurses describe relevant theories on the pathophysiology of delirium. | Very relevant O relevant O less relevant O not relevant O |  |
| General practitioners describe relevant theories on the pathophysiology of delirium. | Very relevant O relevant O less relevant O not relevant O |  |
| Nurses describe predisposing risk factors for the development of delirium. | Very relevant O relevant O less relevant O not relevant O |  |
| General practitioners describe predisposing risk factors for the development of delirium. | Very relevant O relevant O less relevant O not relevant O |  |
| Nurses describe precipitating factors for the development of delirium. | Very relevant O relevant O less relevant O not relevant O |  |
| General practitioners describe precipitating factors for the development of delirium. | Very relevant O relevant O less relevant O not relevant O |  |
| Nurses identify and communicate existing risk profiles and triggering factors for delirium in residents. | Very relevant O relevant O less relevant O not relevant O |  |
| General practitioners identify and communicate existing risk profiles and triggering factors for delirium in residents. | Very relevant O relevant O less relevant O not relevant O |  |
| Nurses initiate case discussions to assess the risk of residents developing delirium. | Very relevant O relevant O less relevant O not relevant O |  |
| General practitioners initiate case discussions to assess the risk of residents developing delirium. | Very relevant O relevant O less relevant O not relevant O |  |
| Nurses recognize that delirium is a multifactorial phenomenon. | Very relevant O relevant O less relevant O not relevant O |  |
| General practitioners recognize that delirium is a multifactorial phenomenon. | Very relevant O relevant O less relevant O not relevant O |  |
| Nurses work collaboratively with other colleagues and across professions. | Very relevant O relevant O less relevant O not relevant O |  |
| General practitioners work collaboratively with other colleagues and across professions. | Very relevant O relevant O less relevant O not relevant O |  |

Module 3

| **Competencies** | **Relevance** | **Comment** |
| --- | --- | --- |
| Nurses know valid screening instruments for assessing delirium. | Very relevant O relevant O less relevant O not relevant O |  |
| Nurses feel confident in their ability to screen for delirium. | Very relevant O relevant O less relevant O not relevant O |  |
| General practitioners know valid diagnostic tools. | Very relevant O relevant O less relevant O not relevant O |  |
| General practitioners diagnose delirium according to the standard diagnostic criteria. | Very relevant O relevant O less relevant O not relevant O |  |
| General practitioners differentiate between various forms of delirium, alcohol withdrawal delirium and other acute neuropsychiatric conditions. | Very relevant O relevant O less relevant O not relevant O |  |
| General practitioners feel confident in their ability to diagnose delirium. | Very relevant O relevant O less relevant O not relevant O |  |
| Nurses describe symptoms of delirium. | Very relevant O relevant O less relevant O not relevant O |  |
| General practitioners describe symptoms of delirium. | Very relevant O relevant O less relevant O not relevant O |  |
| Nurses explain why distinguishing among delirium, dementia and depression is a challenge. | Very relevant O relevant O less relevant O not relevant O |  |
| General practitioners explain why distinguishing among delirium, dementia and depression is a challenge. | Very relevant O relevant O less relevant O not relevant O |  |
| Nurses differentiate between subtypes of delirium. | Very relevant O relevant O less relevant O not relevant O |  |
| General practitioners differentiate between subtypes of delirium. | Very relevant O relevant O less relevant O not relevant O |  |
| Nurses identify possible causes of delirium in residents. | Very relevant O relevant O less relevant O not relevant O |  |
| General practitioners identify possible causes of delirium in residents. | Very relevant O relevant O less relevant O not relevant O |  |
| Nurses use screening or diagnostic instruments safely and correctly. | Very relevant O relevant O less relevant O not relevant O |  |
| General practitioners use screening or diagnostic instruments safely and correctly. | Very relevant O relevant O less relevant O not relevant O |  |
| Nurses provide immediate feedback to the multidisciplinary team if delirium is suspected. | Very relevant O relevant O less relevant O not relevant O |  |
| General practitioners provide immediate feedback to the multidisciplinary team if delirium is suspected. | Very relevant O relevant O less relevant O not relevant O |  |
| Nurses recognize delirium as an emergency situation that requires immediate intervention. | Very relevant O relevant O less relevant O not relevant O |  |
| General practitioners recognize delirium as an emergency situation that requires immediate intervention. | Very relevant O relevant O less relevant O not relevant O |  |

Module 4

| **Competencies** | **Relevance** | **Comment** |
| --- | --- | --- |
| Nurses name possible medications that can be given in the acute phase of delirium. | Very relevant O relevant O less relevant O not relevant O |  |
| Nurses request medical assistance if delirium is suspected. | Very relevant O relevant O less relevant O not relevant O |  |
| General practitioners know possible medications and side effects and the dosages that can be given in the acute phase of delirium. | Very relevant O relevant O less relevant O not relevant O |  |
| General practitioners know possible medications, their side effects, and the dosages that can be given in the acute phase of delirium. | Very relevant O relevant O less relevant O not relevant O |  |
| General practitioners demand support from nursing staff in the treatment of existing delirium. | Very relevant O relevant O less relevant O not relevant O |  |
| Nurses describe nonpharmacological measures for the treatment of delirium. | Very relevant O relevant O less relevant O not relevant O |  |
| General practitioners describe nonpharmacological measures for the treatment of delirium. | Very relevant O relevant O less relevant O not relevant O |  |
| Nurses know the key aspects of liability law when dealing with residents with delirium. | Very relevant O relevant O less relevant O not relevant O |  |
| General practitioners know the key aspects of liability law when dealing with residents with delirium. | Very relevant O relevant O less relevant O not relevant O |  |
| Nurses identify and eliminate possible causes of delirium. | Very relevant O relevant O less relevant O not relevant O |  |
| General practitioners identify and eliminate possible causes of delirium. | Very relevant O relevant O less relevant O not relevant O |  |
| Nurses closely accompany and monitor residents with delirium. | Very relevant O relevant O less relevant O not relevant O |  |
| General practitioners closely accompany and monitor residents with delirium. | Very relevant O relevant O less relevant O not relevant O |  |
| Nurses inform and train relatives of affected residents about delirium and accompany them | Very relevant O relevant O less relevant O not relevant O |  |
| General practitioners inform and train relatives of affected residents about delirium and accompany them | Very relevant O relevant O less relevant O not relevant O |  |
| Nurses communicate empathically and sensitively with residents with delirium. | Very relevant O relevant O less relevant O not relevant O |  |
| General practitioners communicate empathically and sensitively with residents with delirium. | Very relevant O relevant O less relevant O not relevant O |  |
| Nurses develop an interprofessional, facility-specific pathway for the treatment of delirium. | Very relevant O relevant O less relevant O not relevant O |  |
| General practitioners develop an interprofessional, facility-specific pathway for the treatment of delirium. | Very relevant O relevant O less relevant O not relevant O |  |
| Nurses weigh up the use of pharmacological measures together with the interprofessional team with regard to potential side effects. | Very relevant O relevant O less relevant O not relevant O |  |
| General practitioners weigh up the use of pharmacological measures together with the interprofessional team with regard to potential side effects. | Very relevant O relevant O less relevant O not relevant O |  |
| Nurses feel competent to treat delirium. | Very relevant O relevant O less relevant O not relevant O |  |
| General practitioners feel competent to treat delirium. | Very relevant O relevant O less relevant O not relevant O |  |
| Nurses see relatives as an important component in the treatment of delirium. | Very relevant O relevant O less relevant O not relevant O |  |
| General practitioners see relatives as an important component in the treatment of delirium. | Very relevant O relevant O less relevant O not relevant O |  |

Module 5

| **Competencies** | **Relevance** | **Comment** |
| --- | --- | --- |
| Nurses use nonpharmacological measures to prevent delirium. | Very relevant O relevant O less relevant O not relevant O |  |
| General practitioners use nonpharmacological measures to prevent delirium. | Very relevant O relevant O less relevant O not relevant O |  |
| Nurses use measures for cognitive stimulation and emotional relief. | Very relevant O relevant O less relevant O not relevant O |  |
| General practitioners use measures for cognitive stimulation and emotional relief. | Very relevant O relevant O less relevant O not relevant O |  |
| Nurses use reorienting measures to shape the environment. | Very relevant O relevant O less relevant O not relevant O |  |
| General practitioners use reorienting measures to shape the environment. | Very relevant O relevant O less relevant O not relevant O |  |
| Nurses initiate a medication review at regular intervals. | Very relevant O relevant O less relevant O not relevant O |  |
| General practitioners initiate a medication review at regular intervals. | Very relevant O relevant O less relevant O not relevant O |  |
| Nurses promote regular contact between residents and their relatives. | Very relevant O relevant O less relevant O not relevant O |  |
| General practitioners promote regular contact between residents and their relatives. | Very relevant O relevant O less relevant O not relevant O |  |
| Nurses communicate actively and openly with residents | Very relevant O relevant O less relevant O not relevant O |  |
| General practitioners communicate actively and openly with residents | Very relevant O relevant O less relevant O not relevant O |  |
| Nurses initiate case discussions and team discussions on residents at risk of delirium. | Very relevant O relevant O less relevant O not relevant O |  |
| General practitioners initiate case discussions and team discussions on residents at risk of delirium. | Very relevant O relevant O less relevant O not relevant O |  |
| Nurses ensure that residents at high risk of delirium are cared for by people they trust. | Very relevant O relevant O less relevant O not relevant O |  |
| General practitioners ensure that residents at high risk of delirium are cared for by people they trust. | Very relevant O relevant O less relevant O not relevant O |  |
| Nurses are sensitized to the fact that prevention is of great importance both individually and economically. | Very relevant O relevant O less relevant O not relevant O |  |
| General practitioners are sensitized to the fact that prevention is of great importance both individually and economically. | Very relevant O relevant O less relevant O not relevant O |  |
| Nurses recognize the importance of communication between nurses and physicians with regard to the prescription of sedatives/neuroleptics. | Very relevant O relevant O less relevant O not relevant O |  |
| General practitioners recognize the importance of communication between nurses and physicians with regard to the prescription of sedatives/neuroleptics. | Very relevant O relevant O less relevant O not relevant O |  |

Module 6

| **Competencies** | **Relevance** | **Comment** |
| --- | --- | --- |
| General practitioners prescribe on-demand medication to reduce the symptom burden in terminal delirium. | Very relevant O relevant O less relevant O not relevant O |  |
| Nurses distinguish a terminal delirium, from a delirium that arises due to an underlying physical cause and must be considered a potential emergency. | Very relevant O relevant O less relevant O not relevant O |  |
| General practitioners distinguish a terminal delirium, from a delirium that arises due to an underlying physical cause and must be considered a potential emergency. | Very relevant O relevant O less relevant O not relevant O |  |
| Nurses know of drugs that can be used to treat terminal delirium with a heavy symptom burden. | Very relevant O relevant O less relevant O not relevant O |  |
| General practitioners know of drugs that can be used to treat terminal delirium with a heavy symptom burden. | Very relevant O relevant O less relevant O not relevant O |  |
| Nurses are familiar with specific symptoms such as positive hallucinations in terminal delirium. | Very relevant O relevant O less relevant O not relevant O |  |
| General practitioners are familiar with specific symptoms such as positive hallucinations in terminal delirium. | Very relevant O relevant O less relevant O not relevant O |  |
| Nurses promote a calm and reorienting environment for residents with terminal delirium. | Very relevant O relevant O less relevant O not relevant O |  |
| General practitioners promote a calm and reorienting environment for residents with terminal delirium. | Very relevant O relevant O less relevant O not relevant O |  |
| Nurses communicate calmly and empathetically with dying residents and ensure continuous care. | Very relevant O relevant O less relevant O not relevant O |  |
| General practitioners communicate calmly and empathetically with dying residents and ensure continuous care. | Very relevant O relevant O less relevant O not relevant O |  |
| Nurses provide appropriate care and training for relatives and see them as an important component in alleviating the symptoms of delirium in dying residents. | Very relevant O relevant O less relevant O not relevant O |  |
| General practitioners provide appropriate care and training for relatives and see them as an important component in alleviating the symptoms of delirium in dying residents. | Very relevant O relevant O less relevant O not relevant O |  |
| Nurses ask about the concerns, problems and needs of the relatives of dying residents in order to address these adequately and sensitively. | Very relevant O relevant O less relevant O not relevant O |  |
| General practitioners ask about the concerns, problems and needs of the relatives of dying residents in order to address these adequately and sensitively. | Very relevant O relevant O less relevant O not relevant O |  |
| Nurses argue that the intensity of interventions should be guided by the dying resident’s subjective level of suffering and possible danger to themselves and others. | Very relevant O relevant O less relevant O not relevant O |  |
| General practitioners argue that the intensity of interventions should be guided by the dying resident’s subjective level of suffering and possible danger to themselves and others of the dying resident. | Very relevant O relevant O less relevant O not relevant O |  |
| Nurses are critical of unnecessary hospital admissions for dying residents. | Very relevant O relevant O less relevant O not relevant O |  |
| General practitioners are critical of unnecessary hospital admissions for dying residents. | Very relevant O relevant O less relevant O not relevant O |  |
| Nurses take a critical stance on the regular administration of psychotropic drugs for terminal delirium. | Very relevant O relevant O less relevant O not relevant O |  |
| General practitioners take a critical stance on the regular administration of psychotropic drugs for terminal delirium. | Very relevant O relevant O less relevant O not relevant O |  |
| Nurses see relatives as an important component in alleviating the symptoms of delirium in dying residents. | Very relevant O relevant O less relevant O not relevant O |  |
| General practitioners see relatives as an important component in alleviating the symptoms of delirium in dying residents. | Very relevant O relevant O less relevant O not relevant O |  |
